# Development of the child’s ego strength scale: an observation-based assessment of the board game behaviors in play therapy in Korea

**DOI:** 10.1186/s13034-021-00369-3

**Published:** 2021-04-17

**Authors:** Ji Yoen Lee, Young-ae Lee, Mee Sook Yoo

**Affiliations:** 1 Graduate School of Psychotherapy, Play Therapy, Sookmyung Women’s University, 100 Cheongpa-ro 47-gil, Youngsan-gu, Seoul, 04310 Korea; 2 Department of Child Welfare and Studies, Sookmyung Women’s University, 100 Cheongpa-ro 47-gil, Youngsan-gu, Seoul, 04310 Korea

**Keywords:** Ego strength, Ego strength scale, Board game attitude, Observation-based assessment, Play therapy, Early school-aged children

## Abstract

**Background:**

The purpose of this study was to develop a scale for assessing children’s ego strength through the observation of children playing board games in a therapeutic setting. Because ego strength is an index of psychosocial health, it is important to assess ego strength in childhood. In particular, children aged 7 to 9 exhibit their ego-strength characteristics in a situation challenged by self-competence due to their latency period. Therapists can identify such ego strength through game behaviors of children aged 7 to 9 in the play therapy setting. Thus, it is needed to develop a scale by selecting game play behaviors that grasp ego-strength.

**Method:**

Data were collected from 127 play therapists and play therapist-supervisors, who observed 468 play therapy sessions and 55 children aged 7–9 who received play therapy in Korea. The scale was created through content validity verification, factor analysis and verification of criterion-related validity.

**Results:**

We generated a Child’s Ego Strength Scale (CESS) consisting of five sub-factors (*Coping Strategy, Cognitive Strategy, Ego Restriction, Interpersonal Functioning, Frustration Tolerance*) through exploratory factor analysis. The scale met the goodness of fit criteria in a confirmatory factor analysis. The analysis of therapy sessions of children with strong and weak ego strength, as identified by play therapists, showed a significant difference between the two groups in all five sub-variables. There was a significant correlation between the CESS scores and scores of ego strength-related variables of the Rorschach scale, indicating good criterion-related validity.

**Conclusion:**

The CESS appears to be a practical method for the assessment of ego strength in the field of child counseling.

**Supplementary Information:**

The online version contains supplementary material available at 10.1186/s13034-021-00369-3.

## Background

In children, *ego strength* is an index of current psychosocial health [1] as well as an indicator of maturity and adaptation in later developmental stages [2, 3]. Therefore, it is necessary to consider ego strength not only in adulthood and adolescence, but also in early school-aged children, when they must use the ego function to adapt to outside influences. Particularly in the field of play therapy, which has been recognized as an effective psychotherapy approach to treating children’s psychological problems since the late-twentieth century [4–6], evaluating the degree of ego strength is an effective method to assist children in the latency period who are experiencing emotional, behavioral, and adaptive problems. An awareness of a child’s ego strength can help a therapist grasp the current adaptive functioning of the child, establish the treatment goal, and verify therapeutic effectiveness.

### Ego strength in children

Childhood is a time when one breaks away from the self-centered thinking of infancy, more realistic thinking becomes possible, and ego strengthens. Such ego function includes the perception and testing of reality, instinctive impulse regulation, satisfying object relations, remembering and synthesizing experiences, and defending against danger [7]. Children with strong ego function will demonstrate flexibility in their lives [8] and display self-confidence and psychological adjustment. However, children with weak ego function can become lethargic in even minor stress events due to a lack of impulse regulation and resistance to anxiety [9], and they can be more likely to face psychopathological diagnosis [10]. Ego function, which helps children effectively cope with adversity and adapt to their environment, is referred to as ego strength [11], and may be classified as either strong or weak [12].

In Erikson’s psychosocial development perspective, ego strength is regarded as a characteristic expressed from birth. Erikson [13] claimed that there are eight distinct and intrinsic ego strengths throughout one’s lifetime: hope, will, purpose, competence, fidelity, love, care, and wisdom. According to Erikson’s theory, people experience the extremes of ego strength and weakness while resolving the crises of each developmental stage over a lifetime [14–16]. This theory has been supported by Johnson, Beebe, Mortimer, and Snyder [17] who demonstrated that adolescents with well-developed ego strength at a previous stage had more positive self-esteem, lower self-derogation and depressive affect, more positive wellbeing, and more positive intrinsic and extrinsic work values than those with less-developed ego strength. Moreover, such individuals were better adapted for school than those with less-developed ego strength. In contrast, those with weak ego strength have been found to become easily frustrated [18] and unable to effectively cope with their problems [19]. Moreover, it has been reported that sixth-grade elementary school students with weak ego strength are more likely to show less moral behaviors than students with strong ego strength [20].

### Play assessment of ego strength for children

Researchers initially discussed the concept of ego strength with a focus on treating patients with psychiatric disorders [21, 22] but gradually expanded it to include the assessment of general adaptive capacity [1]. More recently, definitions of ego strength have been integrated into a type of coping mechanism. Thus, the components of ego strength have been summarized in the following: (1) the ability to withstand difficult situations and emotional tensions, (2) the ability to make reasonable judgments about these situations and to plan and implement solutions to deal with them [22]. Ego strength is each individual’s attitude toward a problem, reaction to frustration, and ability to adapt using one’s own emotions [23], and it is the developmental foundation that supports ongoing changes [24].

There is a need for objective and standardized measures that can be more effectively used in clinical settings to address ego strength [25]. Although the Ego Strength Scale developed by Barron [21] has been commonly used in clinical settings to measure ego strength [22], this scale is limited to use in adults and adolescents, as it was designed by extracting items related to ego strength from the Minnesota Multiphasic Personality Inventory (MMPI). In addition to Barron’s Ego Strength Scale [21], Markstrom et al. [1] developed a Psychosocial Inventory of Ego Strength (PIES) based on Erickson’s theory for adolescents and adults. However, there is no scale for objectively assessing the degree of ego strength of children in the early school-aged period.

A number of researchers from psychoanalytic backgrounds have attempted to develop ways for assessing the ego functions of children through projective techniques [11]. When these projection tests are applied to a child, they have the advantage of enabling the measurement of the emotional area of the child and of predicting potential defects [26, 27]. Although these techniques have benefits, they take a long time to execute and the validity of the test cannot be guaranteed [22, 28].

To solve these problems, we should adapt many child clinicians’ suggestions in developing a new approach of play assessment that can comprehensively evaluate and analyze children’s behavior from a multidimensional perspective [29, 30]. In play assessment, children’s behavior is observed with a focus on a specific area in the context of play; it has been shown to be a reliable observation system recognized for its stability, sensitivity, and validity compared with other psychometric instruments [31]. Assessment based on these play observations can be used effectively, especially in clinical and therapeutic settings that deal with children’s psychological difficulties including ego strength*.*

### The availability of ego strength scale through child’s board game play attitude

Early school-aged children, about 7 to 9 years old, experience a shift in development in which they move away from pretend play areas, which mark the preschool period, and begin to engage in more structured play with rules [32, 33]. Thus, to assess the ego strength of children in this developmental stage, it is necessary to observe their attitudes during game play therapy, which is the most comfortable psychotherapy technique for children at this developmental stage [34]. In particular, board games, unlike regular play, are goal-oriented and competitive, have rules, and require interaction and more cognitive abilities [33]. Therefore, children must actively utilize an ego process based on secondary process thinking when playing board games [35, 36]. As such, therapists can gain information regarding a child’s ego strength by observing children’s game attitudes while playing board games during play therapy.

The primary reason to assess the degree of children’s ego strength through board games is that these games have rules that determine the player’s win or loss. This competitive characteristic of board games is based on a “model of power,” [37] meaning that the balance of power between child and therapist is invariably broken during the therapy process when board games are used. Thus*,* ego strength becomes easier to test in a gaming situation. Then, the therapist can easily observe and understand the child’s unconscious dynamics such as resistance, transference, and countertransference [32, 38, 39].

Because of these projective features, clinicians who actively use games in play therapy have argued they can obtain diagnostically valuable information from a child’s game attitude [40, 41]. For example, Gardner [42] used the game of checkers as a diagnostic and therapeutic tool [36], and Fried [35] used chess as an analysis tool. In addition, Bow and Quinnell [43] identified a number of socio-emotional characteristics such as cognitive strategies, response to feedback, drive for mastery, and a sense of competency, all of which were observable while playing fine motor skill games with children.

Consistent with this theoretical and empirical evidence, Lee [34, 44] has demonstrated that children with weak ego strength have impaired function in areas such as cognitive strategy, frustration tolerance, impulse regulation, willingness to follow rules, and ability to deal with pressure. Moreover, children with weak ego strength experience more ego depletion while playing games, which may increase the likeliness of inhibition impairment [45]. This can negatively affect the child’s cognitive processes and ability to regulate emotions and impulses [46], resulting in decreased predictive ability [47], lowered cognitive performance [48, 49], and weakened self-control strength [50]. These children appear to try to avoid cognitive dissonance and choose an easier cognitive task [49], show increased negative affect [46], and more often express negative feelings. Thus, structured games in play therapy may be a useful tool that may provide the therapist with opportunities to assess the weakness of the child’s ego strength*.*

### Ego strength classifying criteria through child’s board game play attitude

Therapists can obtain a variety of information about ego strength through a child’s attitude while playing a game. First, therapists can grasp the child’s cognitive strategy. This concept includes judgment ability, such as the ability to anticipate what outcome the behavior will yield, logically identify cause and effect, and make concerted decisions [51]. Further, cognitive strategy refers to the ability to think clearly and deliver one’s thoughts to others along with memory, concentration [52, 53], and flexibility [43, 54].

Second, therapists can identify coping ability [55, 56]. Children who are receiving counseling often exhibit various problematic behaviors because they have problems with self-control [46]. Coping ability includes achievement and problem-solving skills, ability to respond to challenges [3], ability to cope flexibly with problems without being overwhelmed [19, 57], locus of control [3, 43], ability to withstand satisfying desires and immediate urges [58], and goal-oriented behavior to achieve desirable outcomes in the long term [59, 60].

Third, therapists can understand children’s frustration tolerance [43, 61]. As games involve rules and competition [62], people who play games experience winning and losing. As capacity for achievement is an important developmental need for children in the latency period, children may be sensitive to wins and losses during games with their therapist. Thus, children with low ego strength are overly immersed in the game’s outcome [63] and have difficulty withstanding frustration and anxiety [9, 40, 52, 53]. Since enduring loss and frustration is important skill needed to accept and face reality [64], ego strength is defined based on frustration tolerance from a perspective focusing on inner ego function.

Fourth, therapists can understand children’s interpersonal functioning. This refers to the ability to form social relationships and maintain emotional stability and a sense of value [25]. A child has the opportunity to express feelings while playing games with the therapist [36] and further develop the therapeutic relationship [65]. Thus, the therapist can grasp a child’s interpersonal patterns through the attitude the child displays while playing the game. Therefore, the child’s reaction to the therapist’s feedback during the game are important observation variables [40]. In addition, how a child asks for necessary help while playing a game [54] is also an important assessment opportunity for therapists.

Finally, the therapist can understand the child’s ego restriction. The ego can avoid unpleasantness by avoiding uncomfortable situations [66, 67]. When experiencing an unpleasant or stressful situation, ego restriction sacrifices a child’s development while controlling this discomfort [66]. When playing a board game, the child views the therapist as being a more competent adversary and can escape from intense discomfort caused by losing the competition rather than anxiety or guilt. However, if the ego is restricted or inflexible, the child may be unable to withstand this discomfort and will ultimately withdraw, resulting in impaired development. Thus, by giving up one area after another, the ego becomes increasingly less tolerant, disinterested, and ultimately resigned to poor achievement.

Given the information that can be learned through game playing, there is a need to be able to assess a child’s ego strength and the corresponding attitudes in the clinical setting in an objective and quantifiable manner. With such a tool, child psychotherapists would be able to assess children’s ego strength more appropriately and objectively. Moreover, with such a scale, child psychotherapists will be able to set appropriate therapy goals and to evaluate the therapeutic process, thus allowing for the effective management of child’s problems. The main outcome of successful psychotherapy is improvement of ego strength and self-integrity [11]. Thus, pre- and post-therapy assessment using such a scale will also help verify therapeutic effectiveness. Children who undergo psychotherapy that includes ego strength assessment using the newly developed scale in this study may be able to endure internal and external conflicts and grow stronger internally by using their internal resources effectively [68] and by learning to “go to pieces without falling apart” [69].

## Method

This study was divided into four stages to develop the Child’s Ego Strength Scale (CESS): an observational assessment of board game behaviors in play therapy (Additional file 1: Table S1). In play therapy sessions with games, various games such as gross-motor and fine-motor skill games (e.g., Football game, Pick Up Sticks, Jenga, Jumping Monkey, and Tumbling Monkey), games of chance (e.g., Trouble game and Snakes and Ladders) strategy games (e.g., UNO, Yut Nori; Korean traditional board game, and Reverse game) and communication board games (Talking Feeling Doing game, Feeling Card game, and What happened in family game) are utilized depending on the choice of children. In the current study, therefore, therapeutic sessions including various types of games were studied. In Stage 1, five factors and 154 items were pooled through literature review and clinical case analysis, which was composed of 172 game play therapy sessions with 35 children aged between 7 and 9 years. In Stage 2, the numbers of factors and items were adjusted to 4 and 55, respectively, and subsequent internal validity verification was conducted through structured interviews with 10 play therapy experts. In Stage 3, the preliminary scale was used to assess one-time individual game play therapy sessions held by a total of 127 play therapists and play therapy experts for the 468 children who ultimately participated. Then, we performed an Exploratory Factor Analysis (EFA) and Confirmatory Factor Analysis (CFA), and based on these results, a total of five factors and 24 items were finally selected. In Stage 4, we analyzed 24 and 63 game play therapy sessions of children with high and low ego strength, respectively, and verified the difference between these two groups in order to test criterion-related validity. In addition, we evaluated the correlation between the scores of the developed scale and the Rorschach tests in 55 children who were receiving play therapy for internalizing and externalizing problems at child counseling centers in XXX area in order to verify concurrent validity.

### Stage 1: Generate item pool

Stage 1 aimed to generate a pool of game play behaviors in which ego strength can be measured. Five factors and 154 items were extracted through literature review and clinical case analysis.

#### Participants

To extract the items through clinical cases, 172 counseling sessions with 35 children aged 7–9 years who used games in play therapy were analyzed. For this study, we collected information on the therapy sessions involving game playing for children who had received or were receiving play therapy from 2014 to 2016 at the counseling center of a university located in the Seoul area. We targeted children in this age range for several reasons. First, this period corresponds to the latency period of development in which children are increasingly aware of logical thinking and reality [70] and Oedipal obsession and accompanying magical thinking are reduced. Thus, children can act according to principles of reality rather than pleasure principles and are typically striving for aggression control and compliance [65]. Children in this period tend to use games more than pretend play as a communication tool during counseling [32–34, 44]. In particular, from the age of 7, there is a tendency to go back and forth between board games and pretend play during play therapy sessions and then gradually switch to board games [32, 34]. Therefore, age range from seven to nine was considered to be appropriate age range for evaluating ego strength through game attitudes.

All game play sessions were video-recorded, and their contents were transcribed verbatim. The chief presenting complaints for each child were externalizing problems, such as attention problems and oppositional behavior, and internalizing problems, such as depression and anxiety. We excluded children who presenting complaints such as autism, developmental delay or psychosis at first because they could not use games therapeutically in play therapy.

#### Procedure and data analysis

Two sources of data were used to extract appropriate items from game playing behavior to measure ego strength: (1) literature review, through which the subcomponents of ego strength were extracted. Studies were retrieved using multiple strategies, namely a search of Korean electronic databases including NDSL, DB-PIA, RISS, KISS, and English electronic databases including DDOD (Digital Dissertation on Demand), PQDT, PsycARTICLES, ProQuest, and Google Scholar using ego strength and game play therapy-related key terms: ego OR ego strength OR game OR play therapy OR game play therapy OR child. Based on the prior research above, a total of 10 factors that can be grasped through the game attitude were identified; reality testing, judgement, frustration tolerance, emotional control, impulse and behavioral control, problem-solving ability, capacity of expression, defense mechanism, ego restriction, and object relations. Next, the definition for each factor was derived.

(2) Clinical case analysis of play therapy. A total of 172 sessions of game play therapy were analyzed to extract the game attitudes appearing in children during the game. To this end, this researcher, who has a play therapy-supervisor certification and 20 years of experience in play therapy, and two other researchers, each with play therapist certifications with 15 and 5 years of experience, respectively, participated in qualitative analysis for item extraction. Researchers derived 300 game attitude items through the transcribed data and recorded video. Then, three researchers discussed to remove repetitive or uncommon items from the 300 items and confirmed a total of 154 items.

In addition, we removed the factors that are considered difficult to observe in the game attitude of the children from the ten factors, and finalized the five factors by combining factors with similar concepts. Because factors overlapping with items exhibited while arranging observable items in game attitudes for 10 factors, we have combined the relevant factors and renamed them as follows; reality testing, judgement, capacity of expression, and defense mechanism into Cognitive Strategies/emotional control, impulse and behavioral control, and problem-solving ability into Coping Strategies. In addition, object relations were named as Interpersonal Functioning by defining as a collective term for interpersonal relations occurring in relationships with the therapists. Finally, the five factors were determined as Cognitive Strategies, Coping Strategies, Frustration Tolerance, Ego Restriction, and Interpersonal Functioning.

#### Results

Five factors were extracted from literature review related to ego strength, and 154 items were extracted through clinical case analysis. Cognitive Strategies consisted of items corresponding to planning, predictive ability, memory, concentration, language skills, and attribution to the win or loss (e.g., “Complains of injustice when he/she loses”). Coping Strategies consisted of items corresponding to the willingness to follow rules, emotional control, impulse regulation, behavioral control, and problem-solving ability (e.g., “Displays flexibility in changing strategy depending on the situation”). Frustration Tolerance consisted of items corresponding to their reactions to a win and loss, accepting a win or loss in the game, ritual behavior, and expressing physical reaction (e.g., “Stops playing after a bit for games with slightly complicated rules”). Ego Restriction consisted of items corresponding to trying, giving up, degree of change, and degree of task performance. (e.g., “Expresses excessive frustration in the style of having lost everything when he/she loses even once”). Interpersonal functioning consisted of items corresponding to reciprocity, independence, receptiveness to feedback, and trust, all of which measured the quality of the relationship with the therapist (e.g., “Can appropriately ask the therapist for help when necessary”).

### Stage 2: Review and revisions of CESS factors and items through content validity verification

In Stage 2, after verifying the appropriateness of the five factors of the CESS, the five factors were confirmed. The number of items extracted was reduced from 154 to 55 through a content validity test.

#### Participants

Content validity tests were performed on the five factors and 154 items extracted from Stage 1. In general, 5 to 10 expert participants are appropriate when verifying content validity process [71]. The participants for Stage 2 included 10 play therapist-supervisors with an average with more than 15 years of clinical experience in play therapy.

#### Procedure and data analysis

For Stage 2, we visited 10 play therapist-supervisors and conducted one-on-one structured interviews. The interview time was approximately 1 h per therapist. We conducted a content validity test of the five CESS factors and 154 items to verify the appropriateness. In order to verify content validity, four essential components (e.g., domain definition, domain representation, domain relevance, and appropriateness of test construction procedure) should be examined [71]. Accordingly, 10 experts reviewed the validity of the factor definition set in Stage 1 and the appropriateness of each item. Next, the 10 clinical experts evaluated the appropriateness of each of 154 items with a 4-point Likert scales rating to finalize the items to be included in each factor.

Each item was scored using a 4-point Likert scale ranging from *strongly disagree* to *strongly agree*. After calculating the content validity index (CVI), items below 0.78 were deleted [72]. CVI for each item (I-CVI) is the proportion of experts who rated each item as 3 or 4 points on 4-point Likert scales [71].

#### Results

Through the qualitative assessment of 10 play therapist-supervisors, it was determined that the factor consists of five and each definition was appropriate. In the qualitative assessment of each item, 10 play therapist-supervisors revealed some commonality between CESS items and factors, necessitating adjustments and deletions. Accordingly, we calculated I-CVI and removed items below 0.78 for assessment of the content validity. Through this process, duplicated items or items that were difficult to measure clearly were removed, reducing them from 154 to 55.

### Stage 3: Factor analysis of the CESS

In this stage, the five factors and 55 items selected as the preliminary content from Stage 2 were ultimately finalized into a model with five factors and 24 items using factor analysis.

#### Participants

For Stage 3, 127 play therapists and play therapist-supervisors who provide counseling in the play therapy field tested the preliminary scale items that were confirmed in Stage 2. There were one-time individual game play therapy sessions held by a total of 127 play therapists and play therapy experts for the 468 children who participated, the subject of analysis in Stage 3. In play therapists and play therapist-supervisors, 40 years old and younger was the most common age for the therapists with 51.2% (*n* = 65) of the total; 76.4% (*n* = 17) had a master’s degree, 55.1% (*n* = 70) had counseling careers of less than five years, and approximately 100 cases of play therapy to date was the most common response with 41.2% (*n* = 52). All participants except one (99.2%, *n* = 126) reported that they were receiving supervision for counseling. In children, the ages of the children in this study were relatively evenly distributed across first (7-year-olds), second (8-year-olds), and third (9-year-olds) grades; 75.9% (*n* = 355) of participating children were boys, and 59.4% (*n* = 278) had externalizing problems as their chief complaint.

Among the children, there were more boys (*n* = 355) than girls (*n* = 113) among the 468 children who participated (Table 2). This gender difference may be attributed to the fact that the participants of this study were children currently receiving play therapy. Studies of Korea’s clinical settings have reported gender difference in base rate of boy-to-girl ratio. For instance, in Choi and Kim’s study [73] of 157 children aged 6 years or older who participated in play therapy, 111 (70.7%) were boys and 46 (29.3%) were girls. In Kim and Lee’s study [74] of the 51 children who were receiving counseling, 38 (74.5%) were boys and 13 (25.5%) were girls, while in Lee and Han’s study [75] of 14 children aged 4 to 9 years at a counseling center, 9 (64.3%) were boys and 5 (35.7%) were girls. As such, the number of boys receiving therapy was significantly higher than the number of girls in most cases, which appears to be reflected in higher number of boys in the current study.

#### Procedure and data analysis

For Stage 3, the participants were asked to select between two and four children aged 7 to 9 years who were judged as having weak ego strength from their clinical cases and were either currently receiving counseling or had been in counseling in the past 6 months. Then, the preliminary scale was used to evaluate the game play attitudes of each participating child. For this study, we contacted play therapists at several clinics via email, phone calls, and direct visit surveys. We explained the research purpose and conducted a survey on play therapists who agreed to participate in the study by sending out a research bulletin via email, online survey (Google Online), and direct visit surveys. The survey took approximately 10–20 min to complete. Data from a total of 127 play therapists and their individual sessions with the 468 children who participated were collected.

Based on the survey results, the following descriptive statistics were performed to analyze the characteristics of the preliminary scale items confirmed in Stage 2. First, mean and standard deviation were calculated to evaluate bias and ratio characteristics of extreme values. Then, kurtosis and skewness were suggested to check the error and understanding level of the items. In addition, the overall correlation coefficient and range of the items were calculated, and the responses and reactions were examined to verify the normality of the data.

Next, Exploratory Factor Analysis (EFA), reliability analysis, and correlation analysis were performed using SPSS 22 to identify the factor components. Finally, Confirmatory Factor Analysis (CFA) was conducted using AMOS to confirm the suitability of the factor structure.

#### Results

*Item finalization through correlation of preliminary items and iterative verification of reliability.* Before analyzing the factor structure of the preliminary scale, we determined the degree of correlation between items by analyzing the overall correlation of each sub-factor and analyzed the internal consistency of the items. Analysis revealed that the correlation of 9 out of 55 items were not statistically significant; thus, the nine items were removed. Afterwards, multiple factor analysis and item analysis were conducted to remove items with low factor loadings and reliability problems and that posed duplication problems in terms of content validity. Thus, 22 problematic items were eliminated based on these criteria. The final selection included 24 items.

*Descriptive statistics of the scale items.* Descriptive statistics were performed on the items to verify the validity of the final scale, and the results are shown in Table 1. Overall, the item scores were evenly distributed from one to five points with a mean at the three-point level. There were no items exceeding ± 3 in skewness, and kurtosis did not exceed ± 7, indicating that the items used in this study had no problem with normality [76, 77].

**Table 1:** Descriptive statistics of game play attitude observation-based ego strength evaluation scale (N = 468)

	Item number	Min	Max	M	SD	Skewness	Kurtosis
Coping strategy	39	1	5	2.96	1.27	− 0.03	− 1.24
	40	0	5	2.35	1.14	0.63	− 0.51
	38	1	5	2.77	1.28	0.17	− 1.19
	27	1	5	2.33	1.28	0.63	− 0.84
	37	1	5	3.21	1.27	− 0.31	− 1.04
	25	1	5	2.92	1.38	0.05	− 1.35
Cognitive strategy	1	1	5	2.70	1.09	0.18	− 1.00
	4	1	5	2.31	0.97	0.42	− 0.65
	6	1	5	2.88	1.03	0.09	− 0.91
	3	1	5	3.03	1.13	− 0.19	− 1.04
	2	1	5	3.12	1.08	− 0.22	− 0.93
	5	1	5	2.82	1.15	0.16	− 1.04
Ego restriction	32	1	5	3.26	1.22	− 0.34	− 0.96
	31	1	5	3.37	1.20	− 0.35	− 0.96
	33	1	5	3.17	1.20	− 0.23	− 1.03
	30	1	5	2.96	1.26	0.03	− 1.19
Interpersonal functioning	51	1	5	2.65	0.92	0.51	− 0.31
	50	1	5	2.95	0.95	0.25	− 0.49
	53	1	5	2.21	0.77	0.46	0.23
	54	1	5	2.88	0.95	0.19	− 0.70
Frustration tolerance	14	1	5	3.72	1.13	− 0.89	− 0.10
	16	1	5	3.66	1.21	− 0.62	− 0.72
	21	1	5	3.55	1.12	− 0.45	− 0.73
	8	1	5	3.29	1.27	− 0.25	− 1.15

*Exploratory factor analysis.* To check the factorability of the measure by testing sampling adequacy, we conducted a preliminary analysis. In the result, KMO was 0.87, indicating a satisfactory selection of variables for factor analysis [78]. In addition, Bartlett’s sphere formation test, which indicates the suitability of the factor analysis model, was χ^2^ = 4975.167 (*p* < 0.01). This indicates both that factor analysis is appropriate and that there is a common factor, meaning that the existing data for evaluating *ego strength* through game play attitude observation were suitable for factor analysis.

**Fig. 1:**
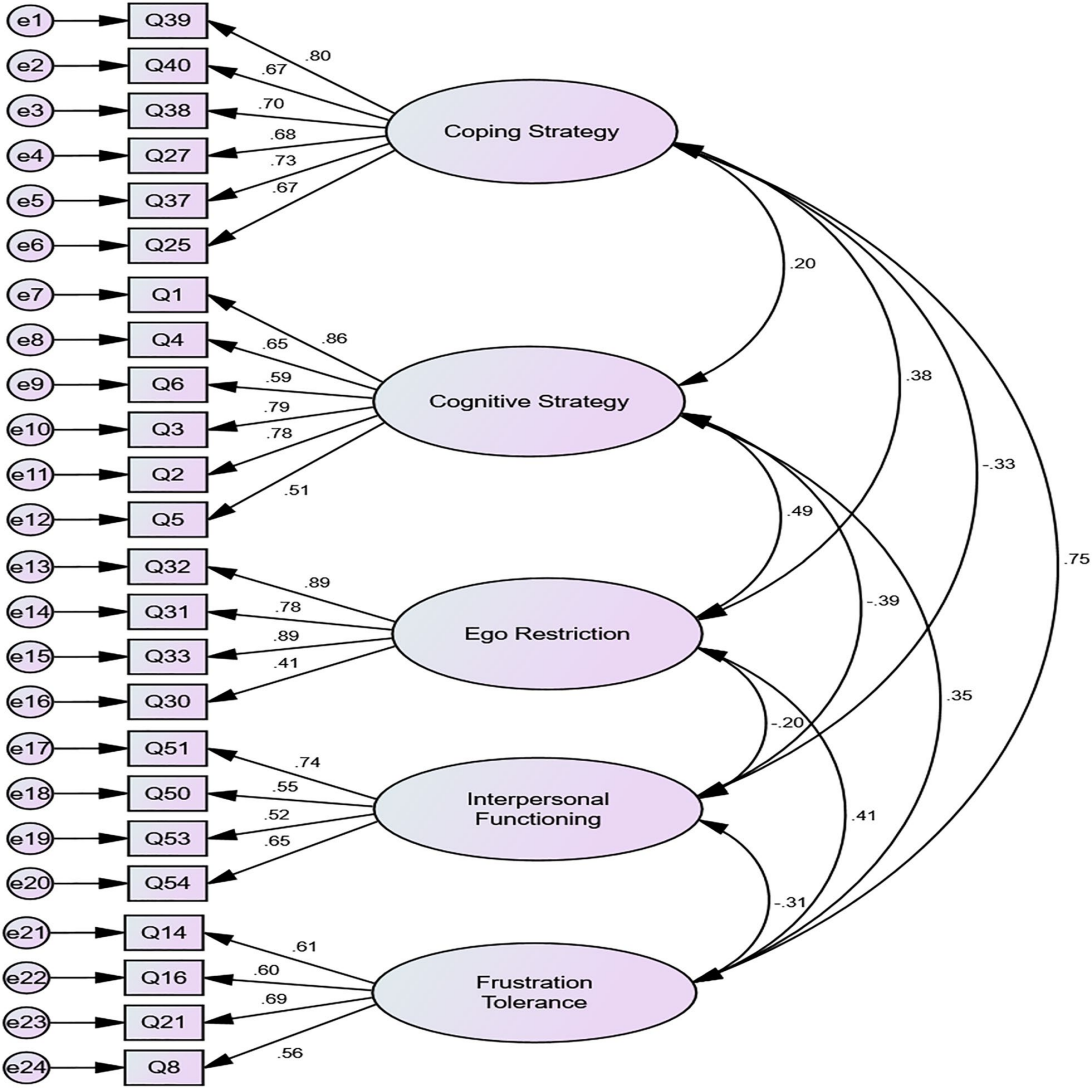
Factor model and standardized factor coefficients through confirmatory factor analysis

To identify the factor structure of the five factors of Coping Strategy, Cognitive Strategy, Ego Restriction, Interpersonal Functioning, and Frustration Tolerance that were theorized in process of the scale development, the principal axis factoring method was applied for EFA. The factorial rotation method used was the varimax technique, which is an orthogonal rotation method. A criterion for the number of factors was based on the Kaiser standard (Eigenvalue > 1.0) and the scree test standard [79, 80]. As shown in Fig. 1 and the results from the EFA presented in Table 2, the items with eigenvalue of 1 or more among the 24 items of the scale were classified into five factors. Factor 1 accounted for 27.8% of the total variance with a factor loading ranging from 0.60 to 0.79, Factor 2 accounted for 13.3% with a factor loading ranging from 0.64 to 0.78, Factor 3 accounted for 8.6% with a factor loading ranging from 0.57 to 0.83, Factor 4 accounted for 6.8% with a factor loading of 0.60 to 0.82, and Factor 5 accounted for 4.6% with a factor loading ranging from 0.50 to 0.72. Thus, the five factors accounted for 61.2% of the total variance. Further, the five extracted factors were consistent with the theoretical concepts selected by the researcher at Stage 1 to measure ego strength through game play attitude observation.

**Table 2:** Exploratory Factor Analysis (N = 468)

Items	Factor loadings					
	F1	F2	F3	F4	F5	Communalities
Coping strategy						
39. Complains of injustice when he/she loses	.79					.67
40. Suspects the therapist of foul play when he/she loses	.74					.61
38. Tries to invalidate the game by saying it was a “practice round” when he/she loses	.73					.57
27. Flips the board and throws the cards or game tools when he/she loses	.72					.58
37. Blames the win or loss on external factors	.69					.59
25. Cries or gets angry when he/she loses in the game a few times because he/she is unable to withstand the loss	.60					.52
Cognitive strategy						
1. Can appropriately use the strategies needed to win		.78				.71
4. Remembers the game rules well		.78				.69
6. Verbally explains the rules of the game appropriately so that the therapist can understand them		.74				.61
3. Moves his/her piece by predicting the therapist’s next behavior		.72				.69
2. Displays flexibility in changing strategy depending on the situation		.69				.66
5. Is not distracted by surrounding stimuli and concentrates on the game		.64				.51
Ego restriction						
32. Says, “I won’t play,” and refuses to begin when listening to the game rules explained and they sound a bit complicated and difficult			.83			.79
31. Does not choose games that have slightly complicated rules			.83			.77
33. Stops playing after a bit for games with slightly complicated rules			.81			.79
30. Tries to play alone for games he/she is not good at and with the therapist for games he/she is good at			.57			.48
Interpersonal functioning						
51. Causes therapist to feel that he/she is interacting with the child when playing games with him/her				.82		.69
50. Can appropriately ask the therapist for help when necessary				.69		.50
53. Enjoys playing games with the therapist				.64		.52
54. Is receptive to the therapist’s feedback when playing the game				.60		.58
Frustration tolerance						
14. Expresses tension as a behavior when he/she seems to be losing					.72	.59
16. Expresses excessive frustration in the style of having lost everything when he/she loses even once					.66	.59
21. Waits patiently for the therapist without prodding when the therapist spends a bit of time thinking during his/her turn					.52	.51
8. Does not stop playing and finishes the game even if he/she seems to be losing					.50	.49
Eigenvalue	6.67	3.20	2.05	1.62	1.14	–
Explained variance %	27.80	13.34	8.55	6.76	4.75	–
Cumulative explained variance %	27.80	41.14	6.76	56.46	61.21	–

*Confirmatory factor analysis.* Next, CFA was conducted to examine whether the items and factors obtained through EFA fit the theoretical model. All scores were greater than 0.90 were considered to indicate a good fit, χ^2^ = 1172.504, *p* < 0.01, goodness of fit index indicator (GFI) = 0.944, Tucker-Lewis Index (TLI) = 0.945, and comparative fit index (CFI) = 0.965 [81, 82]. The root mean square error of approximation (RMSEA) = 0.048 was less than 0.08; based on the fitness index criterion, this factor structure can be evaluated as having good fit [83–86]. In addition, the average variance extraction value was above 0.5, and the conceptual reliability value was above 0.7, verifying the validity of all sub-factors. The factor structure model of this scale is shown in Fig. 1.

*Correlation and reliability of game play attitude observation-based ego strength evaluation scale*. The correlation between the whole scale and each sub-factor and the reliability are shown in Table 3. Each of five factors had a correlation ranging between 0.20 and 0.74 (for all correlations, *p* < 0.01) and was found to have a statistically significant positive correlation. The correlation between the overall scale and each sub-factor ranged between 0.51 and 0.74 (for all correlations, *p* < 0.01). Thus, all showed statistically significant positive correlation. These results show that each sub-factor measures a different domain of ego strength while simultaneously measuring one overarching super-concept. In addition, the Cronbach’s alphas were 0.84, 0.86, 0.85, 0.82, 0.70, and 0.70 for the whole scale, Coping Strategy, Cognitive Strategy, Ego Restriction, Interpersonal Functioning, and Frustration Tolerance, respectively. These results show that this scale has strong internal consistency among the items. In addition, the correlation coefficients between the sub-factors (*r*s ranging from 0.20 to 0.56) were lower than those between the whole scale and sub-factors (*r*s ranging from 0.51 to 0.74). These results show that the sub-factors constituting the scale developed in this study satisfy the criteria that they should be homogeneous yet somewhat independent [87].

**Table 3:** Intercorrelations between factors and internal consistencies (N = 468)

	F1	F2	F3	F4	F5	Total	Cronbach’s α
F1	1						.86
F2	.20^**^	1					.85
F3	.39^**^	.42^**^	1				.82
F4	.26^**^	.31^**^	.18^**^	1			.70
F5	.56^**^	.32^**^	.35^**^	.22^**^	1		.70
Total	.74^**^	.69^**^	.71^**^	.51^**^	.72^**^	1	.84
*M*	13.62	16.87	12.76	10.69	14.21	68.16	–
*SD*	4.89	4.89	3.94	2.63	3.43	13.56	–

M: Mean; SD: standard deviation; F1: coping strategy; F2: cognitive strategy; F3: ego restriction; F4: interpersonal functioning; F5: frustration tolerance; Total: total scale score

^*^p < .05, **p < .01

### Stage 4: Verification of criterion-related validity

#### Participants

Participants in Stage 4 included 25 play therapists and play therapist-supervisors who each had more than 10 years of clinical experience. To verify the criterion-related validity of this scale, 25 participants were each asked to select two children aged 7 to 9 years from their clinical cases, one should be judged to have strong ego strength, the other should be judged to have weak ego strength. These two children were either currently receiving counseling or had received counseling within the past 6 months. Of the 89 sessions with children that were analyzed, there were 24 game play therapy sessions of children evaluated to have strong ego strength and 63 game play therapy sessions of children evaluated to have weak ego strength.

In addition, 55 children aged between 7 and 9 years who received play therapy at two child counseling centers in Seoul area had their game playing attitudes analyzed to verify the scale’s concurrent validity. Children aged 7, 8, and 9 years were 30.9% (*N* = 17), 41.8% (*N* = 23), and 27.3% (*N* = 15), respectively, while 72.7% (*N* = 40) were boys and 27.3% (*N* = 15) were girls.

#### Measurements

The Rorschach test [88] measures the respondent’s cognitive style and psychodynamic construct based on the respondent’s response to ten inkblot cards. The evaluator directly assesses the child’s personality characteristics on the types of variables of the comprehensive system of this test, including resistance to stress, affect control, interpersonal skills, egocentricity, cognitive organization skills, copying deficit skill, presence of depression and perception disorder, excessive vigilance, and degree of compulsion [89]. The Rorschach test provides an optimal opportunity to measure ego impairment because it induces the respondent to use cognitive, affective, and human or representational resources to organize an ambiguous and complex task [90]. Thus, a number of researchers have verified the diagnostic efficacy and validity of the special indexes of the Rorschach CS [91, 92], and contributed to measuring ego strength by using certain variables of the Rorschach test or developing Rorschach Prognostic Scale (RPRS) and Ego Impairment Index (EII) [93, 94].

In our study, the reasons for using the Rorschach test as a criterion-related validity test are as follows. First, it was difficult to ensure the reliability of self-report test due to the age of child participants, thus a type of test conducted by the experimenter was needed. Unlike other self-report tests, the Rorschach test’s responses cannot be consciously manipulated by the test subjects. Second, the validity and reliability of the Rorschach test were scientifically assured with the introduction of Exner’s Comprehensive System (CS) [91, 92]. In addition, the Structural Summary in CS allows the evaluation of the overall domain of individual thinking, emotions, interpersonal relationships, frustration tolerance and coping resources, and cognitive processing and intervention [88], and it was most consistent with the construct of ego-strength that we attempted to evaluate. Past studies have created scales with some Rorschach variables [95], in the current study, however, we excluded random selection of variables and used all seven core sections and six special indexes to evaluate a broader and more comprehensive domain related to ego-strength.

#### Procedure and data analysis

The CESS was designed in a way such that higher scores are associated with weaker ego strength in order to improve clinical efficacy in detecting problematic behaviors. Thus, two positive items from F2: Cognitive Strategy, F4: Interpersonal Functioning, and F5: Frustration Tolerance were reverse scored. First, the participating children undergoing therapy were divided into strong ego strength and weak ego strength groups, and a t-test was performed to analyze differences in scores of the five factors of the CESS between the two groups in order to determine criterion-related validity of the CESS.

Next, concurrent validity of the CESS was determined by analyzing the correlations between the scores of the 5 factors of the CESS and scores of all variables of six special indexes of Rorschach CS (Perceptual Thinking Index; PTI, Depression Index; DEPI, Copying Deficit Index; CDI, Suicide Constellation; S-CON, Hypervigilance Index; HVI, Obsessive Style Index; OBS) and the seven sections of structural summary (Core Section, Ideation Section, Affect Section, Mediation Section, Processing Section, Interpersonal Section, and Self-Perception Section). Two clinical psychologists interpreted the Rorschach test of 55 children, and the inter-rater reliability was good, with 0.89.

#### Results

*Group differences. *As shown in Table 4, a significant difference between the two groups emerged through the analysis of the game play therapy sessions of children who were reported to have strong and weak ego strength to verify the criterion-related validity. As the CESS is designed in a way that higher scores represent weaker ego strength, a higher score on the CESS indicates weak ego strength. The children reported to have weak ego strength had significantly higher mean scores on the overall scale and in the sub-variables of Coping Strategy, Cognitive Strategy, Ego Restriction, Interpersonal Functioning, and Frustration Tolerance. This suggests that this scale can distinguish between groups with strong and with weak ego strength.

**Table 4:** Verification of differences in scores between strong and weak ego strength groups (N = 89)

	Ego strength (*N* = 24)	Ego weakness (*N* = 63)	Difference
Factor	M (SD)	M (SD)	t
Coping strategy	8.50 (3.62)	16.58 (16.59)	− 6.41***
Cognitive strategy	13.29 (3.86)	18.62 (5.32)	− 4.47***
Ego restriction	5.63 (2.60)	11.70 (5.15)	− 5.51***
Interpersonal functioning	8.00 (2.48)	10.67 (2.49)	− 4.47***
Frustration tolerance	8.04 (3.47)	12.24 (3.14)	− 7.64***
Total Score	43.46 (12.05)	71.81 (14.90)	− 9.16***

*Note*. ***p < .001

*Concurrent validity: correlations between CESS and Rorschach.* In order to determine concurrent validity of the CESS, we analyzed the correlations between the scores of the five factors of the CESS and scores of all variables in the seven sections of the comprehensive system of the Rorschach test (Core Section, Ideation Section, Affect Section, Mediation Section, Processing Section, Interpersonal Section, and Self Perception Section) and six special indexes (Perceptual Thinking Index, PTI; Depression Index, DEPI; Copying Deficit Index, CDI; Suicide Constellation, S-CON; Hypervigilance Index, HVI, and Obsessive Style Index, OBS).

The results are shown in Table 5. Mild to moderate correlations were obtained between the CESS factors and Rorschach variables. With regard to Rorschach index, the correlations between F5 (Frustration Tolerance) and F2 (Cognitive Strategy) factors and the CDI were significantly positive, *r* = 0.33 (*p* = 0.013) and *r* = 0.29 (*p* = 0.033), respectively. Rorschach CDI was calculated based on the individual’s stress tolerance and cognitive resources, mental activity (EA < 6, Passive > Active + 1) and coping resources (AdjustD < 0), interpersonal skills (COP < 2, AG < 2), emotional expression (WSumC < 2.5, Afr < 0.46), and intimacy formation (SumT > 1, Isolate/R > 0.24). An increase in CDI suggests that individuals have low stress tolerance, low coping skills, and passive cognitive performance in social settings [96]. Therefore, the positive correlation between F5 (Frustration Tolerance) and F2 (Cognitive Strategy) of the CESS and CDI indicates the validity of the CESS.

**Table 5:** Pearson correlations between CESS and Rorschach indices (N = 55)

	F1	F2	F3	F4	F5	M	SD
CDI	.02	.29^*^	.15	.24	.33^*^	3.40	1.04
DEP1	.05	.08	.27^*^	.21	.14	3.24	1.15
PTI	.00	− .19	− .07	− .06	− .07	2.11	1.50
S-CON	.23	.03	.11	.16	.12	5.35	1.58
HVI	.17	.20	− .01	− .07	− .13	2.27	1.37
OBS	− .02	.08	− .14	− .11	− .08	0.73	0.71
Number of response	.05	− .03	− .30^*^	− 14	− .09	19.24	8.60
Lambda	− .03	− .20	.14	− .04	.29^*^	2.30	3.00
Pure C	.21	.23	.35^**^	.26	.09	.45	.88
COP	− .15	− .17	− .18	− .27^*^	− .27^*^	.33	.70
Afr	.48^***^	.03	.04	.09	− .03	.44	.15
S	.41^**^	− .07	− .07	− .05	− .05	1.27	1.68
A	.04	− .18	− .34^*^	− .10	− .24	4.05	8.10
Dd	− .12	− .12	− .30^*^	− .28^*^	− .06	2.64	3.66
DQ +	− .10	− .27^*^	− .23	− .20	− .14	3.45	3.34
S-%	.31^*^	.06	.12	.08	.07	0.06	0.11

F1: Coping strategy; F2: cognitive strategy; F3: ego restriction; F4: interpersonal functioning; F5: frustration tolerance; CDI: Copying Deficit Index; DEPI: Depression Index; PTI: Perceptual Thinking Index; S-CON: Suicide Constellation; HVI: Hypervigilance Index; OBS: Obsessive Style Index; Lambda: the ratio of pure F response among the total responses; Pure C: pure color response; COP: Cooperative movement; Afr: affect ratio; S: White space; a: active response; Dd: unusual detail response; DQ+: Developmental quality; S-%: White space distortion

^*^p < .05, **p < .01, ***p < .001

In addition, F3 (Ego Restriction) factor was significantly positively correlated to Depression index (DEPI), *r* = . 27 (*p* = 0.049). In four of the seven criteria of the Rorschach DEPI, specific criteria overlap with withdrawal from social domains along with energy decline, such as domain of egocentricity (3r + (2)/R > 0.44 and Fr + rF = 0), restriction of emotional expression (Afr < 0.46, Blends < 4), increase of isolation index ([Bt + 2 × Cl + Ge + Ls + 2 × Na]/R > 0.24), or restriction of cooperative interaction (COP < 2) [88]. In other words, an increase in the DEPI reflects social contraction and withdrawal along with energy decline. Thus, the positive correlation between DEPI and F3 (Ego Restriction) of the CESS seems to ensure the validity of social avoidance and withdrawal tendency for the CESS F3. These results indicate that weaker ego strength is associated with lower social skills and increased avoidance.

The F1 (Coping Strategy) factor and the other CS variables, Afr (degree of interest in affective stimuli), S (white space response), and S-% (proportion of distorted form that involve use of white space), were also significantly positively correlated, *r* = 0.48 (*p* = 0.000), *r* = 0.41 (*p* = 0.002), and *r* = 0.31 (*p* = 0.023), respectively. These results indicate that children who display greater number of inappropriate coping strategies are more likely to show emotional response, passively express frustration, and lose judgement ability so that they have a distorted perception of a situation when frustrated.

F2 (Cognitive Strategy) factor was significantly negatively correlated with DQ + (Developmental quality; assessment of a person’s ability to analyze and synthesize information) *r* = -−0.27 (*p* = *0.0*48), showing more use of inappropriate cognitive strategies is indicative of lower organizing skills using cognitive resources.

F3 (Ego Restriction) factor was significantly correlated with number of response *r* = − .30 (*p* = 0.029), Pure C (number of pure color response) *r* = 0.35 (*p* = 0.009), a (number of flexibility responses) *r* = − .34, (*p* = 0.012), and Dd (unusual detail response) *r* = − .30, (*p* = 0.026). These results show children with high degree of ego restriction are unable to actively express the characteristics of their experience and are likely to have impulsive emotional response.

F4 (Interpersonal functioning) factor was significantly correlated with COP (cooperative movement) *r* = − .27, (*p* ≤ 0.043) and Dd (unusual detail response) *r* = − .28 (*p* = .042). This demonstrates that children who show behaviors that are more inappropriate in interpersonal functioning are less able to positively interpret interactions with others and have poor sensitivity, such as difficulty detecting small details. F5 (Frustration Tolerance) factor was significantly correlated with Lambda (crude index of responsiveness) *r* =  .27 (*p* = .036) and COP (cooperative movement) *r* = − .27 (*p* = .044). This shows that children with lower frustration tolerance are less able to utilize various resources in the environment and experience positive interaction with others [88].

## Discussion

This article describes the empirical procedures employed in developing and evaluating the CESS, a scale designed to assess children’s ego strength by observing and rating their attitudes while they are playing board games in a therapeutic setting.

### Factors and components of items

As the establishment of a stable factor structure plays a very important role in the validation process [97], this study underwent several stages of development. Through this process, we developed the CESS, which consists of five factors and 24 items, and tested its reliability and validity.

The content validity of the CESS was achieved by (a) examining existing scales and both domestic and foreign literature on ego strength, (b) qualitatively analyzing video recording and transcripts of the 172 sessions in game play therapy to generate five sub-factors and 154 preliminary items, and (c). We reviewed qualitatively domain definition, domain representation, domain relevance, and appropriateness of test construction procedure to verify content validity as well as used I-CVI for quantification of content validity of the pre-extracted items. As a result, we generated a final preliminary scale comprising five factors of Coping Strategy, Cognitive Strategy, Ego Restriction, Interpersonal Functioning, Frustration Tolerance and 55 items.

Second, with regard to factor structure validity, we asked 127 play therapists and play therapist-supervisors to assess the game play attitudes of children evaluated as having weak ego strength among the children counseled in the past 6 months using the preliminary scale. We then analyzed data from one-time individual game play therapy sessions for the 468 children to identify the factor structure of the scale. Through an item analysis, nine items without statistically significant item correlation were deleted, and, after deleting 22 additional items with low factor loadings and reliability problems through several factor analyses, 24 items were finally selected. Thus, the five factors were labeled *Coping Strategy, Cognitive Strategy, Ego Restriction, Interpersonal Functioning, and Frustration Tolerance.* An exploratory factor analysis and a confirmatory factor analysis revealed that the CESS was appropriately composed of items measuring the five constructs and that each factor measured one unified construct of ego strength.

### Validity and reliability of CESS

There was significant correlation between the game play behavior observation-based CESS and the sub-factors in terms of construct validity. We also analyzed 89 game play therapy sessions of children with strong and weak ego strength identified by 24 play therapy specialists and verified the between-groups difference. There was significant difference between the two groups both on their overall scale scores and in all five factors: *Coping Strategy, Cognitive Strategy, Ego Restriction, Interpersonal functioning, and Frustration Tolerance*.

The concurrent validity of the CESS was determined by analyzing the correlation between the CESS score and Rorschach Comprehensive System (Rorschach CS) scores.

The results showed that of the five factors of the CESS, the frustration tolerance and cognitive strategy factors were significantly positively correlated with copying deficit index (CDI). An increase in CDI score particularly indicates impairment of the interpersonal skills and social comfort that are needed for problem solving [88]. The findings of this study illustrated that children with low frustration tolerance and poor cognitive strategies have insufficient resources to deal with stress and impaired coping skills.

It is noteworthy that the results of this study showed significant correlations between two of the Rorschach CS sub-factors of ego strength and the coping impairment index. This result indicates that the construct of this scale, which measures ego strength, is valid based on the exiting theory defining ego strength as a coping mechanism [22]. Moreover, the ego restriction factor of the CESS was significantly and positively correlated with the Depression index (DEPI) on the Rorschach CS. According to Exner [88], high DEPI is indicative of individuals with depressed or unstable affect and a tendency to be self-absorbed and avoidant. In this study, children with greater degree of ego restriction were less able to respond actively to the environment and showed avoidance due to lowered energy level.

Other findings from the present study indicated that the children who displayed a greater number of inappropriate coping strategies were more likely to show emotional responses and lost the ability to make reasonable judgements when they were angry. The children with a greater number of inappropriate cognitive strategies had lower cognitive organizational skills, whereas those with greater degree of ego restriction were unable actively to express their experiences and had a tendency to show impulsive emotional responses. In addition, lower interpersonal functioning and frustration tolerance were indicative of the inability to cooperate with others and to use available resources from the environment due to the lack of sensitivity [88]. Taken together, these results suggest that the CESS may be a useful tool in measuring ego strength.

The results of the scale reliability verification indicated that Cronbach’s alpha was 0.84 for the whole scale and 0.70 to 0.86 for the sub-factors. As described above, this scale can be regarded as an instrument with relatively high reliability and validity as well as an observational tool that consists of items and factors appropriate for measuring ego strength in children based upon game attitudes.

### Strengths and limitations

The study’s main strength is its development of a game play assessment tool for child patients. As many researchers and clinicians have suggested that children’s ego strength can be assessed by observing game attitudes, this study is meaningful in that it developed this as a measurable scale. In fact, this study began with the practical need of researchers, who have 20 to 35 years of clinical experience in play therapy. Through this clinical experience, we could perform the practical task of selecting meaningful items through video and conducting the verbatim analysis of the children’s game play therapy scenes as well as an extensive literature review. A long clinical experience served as a basis for item extraction and selection and allowed us to develop a more realistic scale for clinical use.

Therefore, when using this scale, therapists can observe children’s game attitudes more systematically and specifically in terms of ego strength and work to objectify them. This is expected to contribute to case formulation and therapists’ goal setting for children in counseling, as its therapeutic effectiveness can also be verified. Board games are commonly used by children counselors in elementary schools in Korea. Thus, use of this scale in school counseling settings may aid school counselors to identify children’s ego strength and developing a profile of strengths and weaknesses using each of the sub-factors.

In addition, therapists who do not actively use board games in therapeutic settings would benefit from using this assessment prior to using play therapy.

The current study has the following limitations. First, the subject of this study, play therapy sessions using games, include 127 play therapists’ responses to the sessions of 3 or 4 child clients. Thus, there is a possibility to have some dependency in the data. Also, it is possible that there is some bias for therapists in remembering specifically about the child’s game attitude by recalling their therapy sessions within 6 months.

Second, although many behaviors observed in the clinical setting were extracted to develop this scale, most were ultimately deleted in the final item selection process. However, some of the deleted items may measure children’s *ego strength* more specifically. Thus, additional research is needed to continuously test the validity of the scale through the process of selecting and revising the scale with more specific and accurate items to assess children’s ego strength.

Third, the results of this study are limited to narrow age groups as the children analyzed in this study were aged 7 to 9 years who were receiving play therapy in Korea that utilizes board games as a therapeutic tool. Although this age range was chosen because children aged 7 to 9 are at the most appropriate developmental phase to measure the child’s ego strength through the observation their game attitudes, future study is needed to verify the reliability of the CESS in children aged 10 years or older.

Fourth, there was a difference in the number of boys and girls in the samples of this study, as boys are significantly more likely to be referred to counseling than girls in Korea. Thus, a future research that considers gender and presenting symptoms is needed.

Lastly, child clients used a variety of board games during the play therapy sessions in our study. These different games may cause additional variance related to differences between games.

## Conclusion

CESS is a play assessment scale of child’s board game behaviors for ego strength. Our study is consistent with previous research suggesting that therapists can easily observe a child’s unconscious dynamics through the child’s game play behavior [32, 38, 39]. Especially in Korea, it is emphasized to complete a given task successfully for children due to the achievement-oriented cultural values [98, 99]. Accordingly, it is possible that Korean children are relatively more sensitive to failure than children from other countries. For Korean children, thus, it is highly likely that the emotional and behavioral characteristics related to victory or defeat would be presented relatively clear when playing games about winning or losing. Considering that these cultural characteristics of Korean children are frequently observed in therapeutic settings, this study was designed for practical need to evaluate children’s ego strength through games, establish treatment goals and plans based on the assessment of ego strength, and eventually support children in need.

It is important to note that coping strategy was the first among the sub-factors of this scale. This is consistent with the integration of the ego strength concept with coping mechanism, a shift from its initial pathologic implication [22]. One point to consider in the study results is that ego restriction was included in CESS. Ego restriction is caused by external stimuli such as competition failure, unlike denial and avoidance, which are used to escape internal stimuli such as anxiety or guilt. Therefore, the result of this study that a defense mechanism of ego restriction can be observed in a child when playing a game is noteworthy in therapy session. CESS consists of conscious and unconscious factors that represent the ego strength of children, and we believe that CESS may useful to assess the ego strength of child patients in play therapy sessions.

## Supplementary Information

**Additional file 1: Table S1.** Child’s ego strength scale form: An observation-based assessment of the board game behaviors in play therapy.

## Data Availability

The datasets generated and/or analysed during the current study are not publicly available because of individual privacy considerations and limitations from ethics approval. The datasets are available from the corresponding author on reasonable request. Note that verbatim-transcribed data are in Korean.

## Abbreviations

CESS: Child’s Ego Strength Scale
I-CVI: Item level-Content Validity Index
Rorschach CS: Rorschach Comprehensive System
RPRS: Rorschach Prognostic Scale
EII: Ego Impairment Index
PTI: Perceptual Thinking Index
DEPI: Depression Index
CDI: Copying Deficit Index
S-CON: Suicide Constellation
HVI: Hypervigilance Index
OBS: Obsessive Style Index
DQ+: Developmental quality
Pure C: Number of pure color response
Dd: Unusual detail response
COP: Cooperative movement
Afr: Degree of interest in affective stimuli
S: White space response
S-%: Proportion of distorted form that involve use of white space
CDI: Copying deficit index
