# Supplementary material for: Development of the child’s ego strength scale: an observation-based assessment of the board game behaviors in play therapy in Korea
Source: Child Adolesc Psychiatry Ment Health. 2021 Apr 17;15:20. doi: 10.1186/s13034-021-00369-3 (PMC8053275; doi:10.1186/s13034-021-00369-3)
Supplement: Supplementary file 1 — Additional file 1: Table S1. Child’s ego strength scale form: An observation-based assessment of the board game behaviors in play therapy. [file 13034_2021_369_MOESM1_ESM.docx]

**Supplementary material**

**for**

**Development of the child’s ego strength scale: An observation-based assessment of the board game behaviors in play therapy in Korea**

**Supplementary Table S1**

*Child’s Ego Strength Scale Form: An Observation-Based Assessment of the Board Game Behaviors in Play Therapy*

1= Strongly disagree; 2= Disagree, 3= Neither agree nor disagree, 4= Somewhat agree, 5= Strongly agree

| **When playing games during the counseling process, the child…** | | | 1 | 2 | 3 | 4 | 5 |
| --- | --- | --- | --- | --- | --- | --- | --- |
| Coping  Strategy | 39 | complains of injustice when he/she loses  (e.g., “you’ve played this game a lot,” “you’re an adult,” etc.) |  |  |  |  |  |
|  | 40 | suspects the therapist of foul play when he/she loses. |  |  |  |  |  |
|  | 38 | tries to invalidate the game by saying it was a “practice round” when he/she loses |  |  |  |  |  |
|  | 27 | flips the board and throws the cards or game tools when he/she loses. |  |  |  |  |  |
|  | 37 | blames the win or loss on external factors. (e.g., “because of the color of the game piece,” “because of the therapist,” etc.) |  |  |  |  |  |
|  | 25 | cries or gets angry when he/she loses in the game a few times because he/she is unable to withstand the loss. |  |  |  |  |  |
| Cognitive  Strategy | 1 | can appropriately use the strategies needed to win. |  |  |  |  |  |
|  | 4 | remembers the game rules well. |  |  |  |  |  |
|  | 6 | verbally explains the rules of the game appropriately so that the therapist can understand them. |  |  |  |  |  |
|  | 3 | moves his/her piece by predicting the therapist’s next behavior. |  |  |  |  |  |
|  | 2 | displays flexibility in changing strategy depending on the situation. |  |  |  |  |  |
|  | 5 | is not distracted by surrounding stimuli and concentrates on the game. |  |  |  |  |  |
| Ego  Restriction | 32 | says, “I won’t play,” and refuses to begin when listening to the game rules explained and they sound a bit complicated and difficult. |  |  |  |  |  |
|  | 31 | does not choose games that have slightly complicated rules. |  |  |  |  |  |
|  | 33 | stops playing after a bit for games with slightly complicated rules. |  |  |  |  |  |
|  | 30 | tries to play alone for games he/she is not good at and with the therapist for games he/she is good at. |  |  |  |  |  |
| Interpersonal  Functioning | 51 | causes therapist to feel that he/she is interacting with the child when playing games with him/her. |  |  |  |  |  |
|  | 50 | can appropriately ask the therapist for help when necessary. |  |  |  |  |  |
|  | 53 | enjoys playing games with the therapist. |  |  |  |  |  |
|  | 54 | is receptive to the therapist’s feedback when playing the game. |  |  |  |  |  |
| Frustration  Tolerance | 14 | expresses tension as a behavior when he/she seems to be losing.  (e.g., hasty behavior, fidgeting, lying on the floor, etc.) |  |  |  |  |  |
|  | 16 | expresses excessive frustration in the style of having lost everything when he/she loses even once. |  |  |  |  |  |
|  | 21 | waits patiently for the therapist without prodding when the therapist spends a bit of time thinking during his/her turn. |  |  |  |  |  |
|  | 8 | does not stop playing and finishes the game even if he/she seems to be losing. |  |  |  |  |  |
